# *TaUGT6*, a Novel UDP-Glycosyltransferase Gene Enhances the Resistance to FHB and DON Accumulation in Wheat

**DOI:** 10.3389/fpls.2020.574775

**Published:** 2020-10-16

**Authors:** Yi He, Lei Wu, Xiang Liu, Peng Jiang, Lixuan Yu, Jianbo Qiu, Gang Wang, Xu Zhang, Hongxiang Ma

**Affiliations:** ^1^CIMMYT-JAAS Joint Center for Wheat Diseases, Jiangsu Academy of Agricultural Sciences, Nanjing, China; ^2^Tibet Agriculture and Animal Husbandry University, Linzhi, China; ^3^Institute of Food Safety and Nutrition, Jiangsu Academy of Agricultural Sciences, Nanjing, China; ^4^Jiangsu Co-Innovation Center for Modern Production Technology of Grain Crops, Yangzhou University, Yangzhou, China

**Keywords:** wheat, UDP-glucosyltransferase, resistance, *Fusarium* head blight, deoxynivalenol

## Abstract

*Fusarium* head blight (FHB), a devastating wheat disease, results in loss of yield and production of mycotoxins including deoxynivalenol (DON) in infected grains. DON is harmful to human and animal health and facilitates the spread of FHB symptoms. Its conversion into DON-3-glucoside (D3G) by UDP-glycosyltransferases (UGTs) is correlated with FHB resistance, and only few gene members in wheat have been investigated. Here, *Fusarium graminearum* and DON-induced *TaUGT6* expression in the resistant cultivar Sumai 3 was cloned and characterized. TaUGT6::GFP was subcellularly located throughout cells. Purified TaUGT6 protein could convert DON into D3G to some extent *in vitro*. Transformation of *TaUGT6* into *Arabidopsis* increased root tolerance when grown on agar plates containing DON. Furthermore, *TaUGT6* overexpression in wheat showed improved resistance to *Fusarium* spread after *F*. *graminearum* inoculation. Overall, this study provides useful insight into a novel *UGT* gene for FHB resistance in wheat.

## Introduction

Wheat (*Triticum aestivum* L.) is the third largest grain crop after corn and rice in terms of production, with > 700 million tons consumed by 2.5 billion people worldwide (Ma et al., 2020). However, wheat production is challenging due to the increasing threat of abiotic and biotic stresses. *Fusarium* head blight (FHB), primarily caused by *Fusarium graminearum*, is one of the most devastating wheat diseases in China and other parts of the world, where frequent rainfall occurs from the flowering to early grain filling stages (Ma et al., 2019). This disease can cause marked yield loss via poor grain filling and reduced test weight, with up to 70% yield losses under favorable conditions (Bai et al., 2000). More importantly, FHB-infected grains may contain high concentrations of mycotoxins such as deoxynivalenol (DON) produced by *Fusarium* species, which are harmful to human beings and animals and have become an even more critical public concern (Ma et al., 2019). Furthermore, DON is considered a virulence factor that induces FHB spread within a spike, and DON delivery to the rachis node could be a trigger for biotrophy–necrotrophy switch (Lemmens et al., 2005; Bönnighausen et al., 2019).

*Fusarium* head blight resistance is a complex quantitative trait controlled by multiple quantitative trait loci (QTLs), and approximately 500 QTLs have been identified since 1999 when the first FHB resistance QTL was published for wheat (Bai et al., 1999; Waldron et al., 1999; Buerstmayr et al., 2019). Five FHB-resistant QTL types were reported in wheat, e.g., type I representing resistance to initial infection, type II representing resistance to fungal spread, and type III representing resistance to mycotoxin accumulation (Schroeder and Christensen, 1963; Mesterházy, 1995). *Fhb1*, a major QTL for type II resistance, is the most comprehensively studied region to date and was cloned by different groups (Rawat et al., 2016; Li et al., 2019; Su et al., 2019). However, QTLs related to other resistance types have not been fully elucidated, and only few type III resistance QTLs have been tested for their ability to detoxify DON or enhance resistance to this toxin (Gunupuru et al., 2017). Cloning genes related to DON resistance or detoxification of transgenic or gene-edited crops may provide an important method to improve FHB resistance in wheat.

Deoxynivalenol is an aggressiveness factor with an important role in spike and seed colonization, and its detoxification will help reduce pathogen virulence and increase FHB resistance, leading to fewer symptoms related to DON-susceptible wheat cultivars (Lemmens et al., 2005). Many genes reportedly affect DON resistance in a direct manner, including genes encoding transporters, detoxification enzymes, and regulators of hormone signaling (Gunupuru et al., 2017). The ABC transporters *PDR5* and *TaABCC3.1* were reported to contribute to DON tolerance (Mitterbauer and Adam, 2002; Walter et al., 2015). The maintenance of hormone homeostasis may contribute to DON tolerance. Silencing of the *ethylene insensitive 2* gene in wheat resulted in FHB resistance and gain of function related to gibberellic acid-sensitive DELLA NIL lines that showed more resistance to the *Fusarium*-infected spikes and reduced DON-induced cell death rate (Chen et al., 2009; Saville et al., 2012). Enzymes involved in diverse processes have been shown to enhance DON resistance, e.g., the wheat cytochrome P450 TaCYP72A, which contributes to DON host resistance and bacterial cytochrome P450 *Ddna*, which can convert DON to 16-hydroxy-DON (Ito et al., 2013; Gunupuru et al., 2018). UDP-glycosyltransferases (UGTs) are the most widely studied enzymes that have been shown to confer DON and FHB resistance via conjugation of DON to DON-3-O-glucoside (D3G).

UDP-glycosyltransferases are a multigenic and highly divergent superfamily of enzymes widely found in all living organisms, many of which share a close relationship with disease resistance (Gachon et al., 2005). The C-terminus of UGTs contains a conserved region of 44 amino acids, which is termed plant secondary product glycosyltransferase (PSPG) (Vogt and Jones, 2000). Many UGTs were found based on this conserved region such as > 100 UGTs found in wheat (He et al., 2018). The first identified UGT capable of detoxifying DON was DOGT1, which catalyzed the transfer of glucose to DON, creating a less toxic D3G, and overexpressing this gene results in increased DON tolerance in *Arabidopsis* (Poppenberger et al., 2003). Moreover, other UGTs have been reported to be involved in DON tolerance or FHB resistance. Barley *HvUGT13248* is highly induced by DON and can convert DON to D3G, and the overexpression of this gene in *Arabidopsis* and wheat reduced sensitivity to DON and FHB spread (Schweiger et al., 2010; Li et al., 2015). Two homologs of *HvUGT13248* were found in *Brachypodium distachyon*, and overexpression of *Bradi5g03300* increased root tolerance to DON and enhanced FHB resistance (Schweiger et al., 2013a; Pasquet et al., 2016). *HvUGT-10W1* from an FHB-resistant barley cultivar also contributes to FHB resistance (Xing et al., 2017). In wheat, only few *UGT* genes have been identified. *TaUGT1*, *TaUGT2*, and *TaUGT4* were only analyzed at the transcriptional level following *F*. *graminearum* infection and were not further characterized (Lin et al., 2008; Ma et al., 2015). *TaUGT12887/TraesCS5B02G148300* conferred increased DON resistance when expressed in yeast (Schweiger et al., 2013b). *TaUGT3* and *TaUGT5* can enhance DON tolerance when expressed in both *Arabidopsis* and wheat (Ma et al., 2010; Xing et al., 2018; Zhao et al., 2018). The expression of *Traes_2BS_14CA35D5D*, orthologous to *Bradi5g03300*, is highly inducible by DON, and transformation of this *UGT*-encoding gene from wheat into *B*. *distachyon* enhanced FHB resistance and root tolerance to DON (Gatti et al., 2018). In this study, we cloned and characterized a novel *UGT* gene *TaUGT6* involved in resistance to DON tolerance and *Fusarium* spread.

## Materials and Methods

### Plant Materials and Growth Conditions

Sumai 3, Annong 8455, and Fielder were grown in plastic pots or in single-row-plots in greenhouses at campus of Jiangsu Academy of Agricultural Sciences, Nanjing, China. Spikelets at the early anthesis stage were chosen for *F*. *graminearum* or DON inoculation, as described previously (Xu et al., 2004). Ten microliters of *F*. *graminearum* conidia suspension (approximately 5 × 10^5^ conidia spores/mL) were injected into one floret at intermediate positions of spikelets and covered with plastic bags for 3 days to retain moisture. The infected spikelets were harvested at 12, 24, 48, and 72 h after inoculation. During DON inoculation, 5 μL DON (10 mg/L) (Sangon, China) was injected into florets and collected after 2, 4, 8, 12, and 24 h of inoculation. All samples were frozen immediately in liquid nitrogen and stored at -80°C. *A*. *thaliana* were grown in a chamber under a 16/8-h light/dark condition at 21°C.

### RNA Extraction and RT-qPCR

Total RNA was isolated using the Promega SV total RNA isolation system (Promega, United States), and first-strand cDNAs were synthesized using the PrimeScript 1st strand cDNA Synthesis Kit (Takara Bio, Dalian, China). RT-qPCR was performed using a Roche thermal cycler 96 using the SYBR Green reagent (Takara Bio, Dalian, China). *Tubulin* in wheat and *actin* in *A*. *thaliana* were used as internal reference genes. RT-qPCR was performed as described previously (He et al., 2018). Triplicates were applied for all qPCR analyses in this study. Data were calculated using the 2^–ΔCt^ method (Livak and Schmittgen, 2000). The primers used in RT-qPCR are listed in Supplementary Table 1.

### Subcellular Localization of *TaUGT6*

Full-length cDNA of *TaUGT6* was cloned at the Xho I and Spe I sites of the pA7-eGFP plasmid with the primers listed in Supplementary Table 1. The resultant plasmids were coupled with gold particles, bombarded into onion epidermal cells, and observed as previously described (Scott, 1999). Fluorescence signals of green fluorescent protein (GFP) were observed under UltraView VOX (PE, United States).

### Sequence Alignment and Phylogenetic Analysis

TaUGT6 and several other UGT proteins (TaUGT1, TaUGT2, TaUGT3, TaUGT4, TaUGT5, HvUGT13248, Bradi5g03300, and DOGT1) that had been previously reported to contribute to FHB resistance were selected for alignment by DNAMAN using the pairwise method (Poppenberger et al., 2003; Lin et al., 2008; Ma et al., 2015; Pasquet et al., 2016; Li et al., 2017; Xing et al., 2018; Zhao et al., 2018). BLAST analysis of the full-length amino acid sequences of TaUGT6 was performed to identify close homologs of TaUGT6 in the Phytozome database^1^. Multiple sequence alignment of UGT protein sequences was performed by MUSCLE^2^ and adjusted manually using GeneDoc^3^. Neighbor-joining trees based on the full-length UGT protein sequences were constructed using MEGA 7.0^4^ with the following parameters: Poisson correction, pairwise deletion, and bootstrap (1000 replicates; random seed).

### Glucosyltransferase Activity Assay

Full-length cDNA of *TaUGT6* was cloned into a prokaryote expression plasmid pGEX-4T-1, and the GST-tagged TaUGT6 recombinant protein was expressed in *E*. *coli* and purified using affinity chromatography (Liu et al., 2020). Then, glucosyltransferase activity assay was performed using a reaction mix (200 μL) containing 20 μL of 0.5 M Tris-HCl (pH = 7.0), 10 μL of 50 mM MgSO4, 10 μL of 200 mM KCl, 5 μL of 0.1 M UDP-glucose, 2 μL of 10% (v/v) β-mercaptoethanol, 5 μL of 10 mM DON, and 2 μg of the recombinant protein. Following incubation for 3 h at 30°C, 20 μL of 240 mg/ml trichloroacetic acid solution was added to the reaction mix. Then, the reaction mix was filtered with a 0.22-μm membrane, frozen immediately in liquid nitrogen, and stored at −80°C until used for high-pressure liquid chromatography/electrospray ionization–tandem mass spectrometry (HPLC-MS/MS).

### Generation of Transgenic *A. Thaliana* and DON Resistance Evaluation

*TaUGT6* sequence was amplified using the primers listed in Supplementary Table S1 and was cloned into plant vector pHB with the CaMV35S promoter. pHB–*TaUGT6* was transformed into the *Agrobacterium tumefaciens* strain GV3101 and then into the WT Col-0 via the floral dip method (Clough and Bent, 1998). Transgenic plants were selected through hygromycin resistance evaluation and PCR. The independent T3 homozygous lines TaUGT6-2, TaUGT6-6, and Col-0 seeds were sterilized and placed on 1/2 MS solid medium for a 2-day dark treatment at 4°C to synchronize germination and then for 3 days in a chamber under a 16/8-h light/dark cycle at 21°C. The seedlings with the same size and growth vigor were transferred to 1/2 MS media containing different concentrations of DON (5 and 30 ppm). Seedlings under 5 and 30 ppm DON treatments were grown for 7 and 21 days, respectively, under identical environmental conditions before the phenotype was documented. The longest root of each seedling was recorded, and the significant level of data was analyzed using Student’s *t*-test.

### Generation of Transgenic Wheat and FHB Resistance Evaluation

*TaUGT6* was cloned into the vector pTCK303 with maize ubiquitin promoter. pTCK303–*TaUGT6* was transformed into the *A*. *tumefaciens* strain EHA105 and then into Fielder, which was highly susceptible to FHB by the *Agrobacterium*-mediated method (Zhang et al., 2018). Transgenic plants were selected through hygromycin resistance evaluation and PCR. The independent T3 homozygous lines TaUGT6-779, TaUGT6-790, and Fielder were grown in plastic pots or in single-row-plots in greenhouses and spikelets at an early anthesis stage were chosen for *F*. *graminearum* inoculation as described above. FHB severity was determined as PSS every 2 days after 7 dai. For statistical analysis, Student’s *t*-tests were performed to compare each transgenic line with the nontransformed Fielder control.

### Mycotoxin Extraction

Freshly wheat spikes were grounded to powder at 15 dai, shaken with acetonitrile/water (1:1, v/v), which had been treated with 0.1% methanoic acid (ROE, United States). Then, it was centrifuged for 5 min at 3,500 g, and 2 ml of the supernatant was purified with an extraction salt pack (200 mg of magnesium sulfate, 100 mg of sodium citrate, 100 mg C18, and 100 mg of primary secondary amine). After centrifugation, the supernatant was transferred to a new tube and concentrated under nitrogen. Before analysis, the extracts were diluted with 0.6 mL extract solution, filtered using 0.22 μm filters, and then were analyzed by HPLC-MS/MS.

### HPLC-MS/MS

The procedure of the HPLC-MS/MS was performed as described by Qiu et al. (2020). Briefly, these analyses were analyzed with a LC-20ADXR liquid chromatograph (Shimadzu, Japan) coupled to an AB SCIEX TRIPLE QUAD 3500 triple-quadrupole mass-spectrometric detector (Applied Biosystems, United States). A Kinetex 100A C18 column (100 × 4.6 mm, 2.6 μm) from Phenomenex (Torrance, United States) was used. Of each sample, 2 μL was injected into the column, the flow rate was 0.5 mL/min, and the column temperature was maintained at 40°C. The mobile phase consisted of 5 mM ammonium acetate-acetic acid (99.9/0.1, v/v) (A) and methanol (B). Gradient elution was performed with the following conditions: 0–0.01 min with a linear gradient to 10% A; 1.6–2 min with a linear gradient from 10% to 35% A; 2–4.44 min with a linear gradient to 55% A; 4.44–6.5 min with a linear gradient from 55% to 90% A; 6.5–12 min held at 90% A; and 12–15 min, solvent A remained constant at 10%. The mass spectrometer was operated in the following settings: gas temperature, 500°C; gas flow rate, 10 L/min; nebulizer gas pressure, 50 psi; and capillary voltage, 5500 V. Nitrogen was used as the ion source and the collision cell. Mycotoxins were analyzed via multiple reaction monitoring (MRM). The detection was made by monitoring the most intensive precursor to fragment transitions at mass-to-charge ratio (m/z) 297.200 to 203.100 for DON under positive mode, and m/z 503.100 to 427.100 for D3G under negative mode. The DON (Sigma-Aldrich, United States) and D3G (Sigma-Aldrich, United States) were used as standards.

### Scanning Electron Microscopy

The infected spikelets at 3 days after inoculation (dai) were fixed in 3 % glutardialdehyde for 2 h, dehydrated in a graded alcohol series, coated with gold and examined using Zeiss EVO LS10 scanning electron microscope (Carl Zeiss, Germany).

## Results

### Inducible Expression of *TaUGT6* by *F. graminearum* and DON in the FHB-Resistant Wheat Cultivar Sumai 3

**FIGURE 1:**
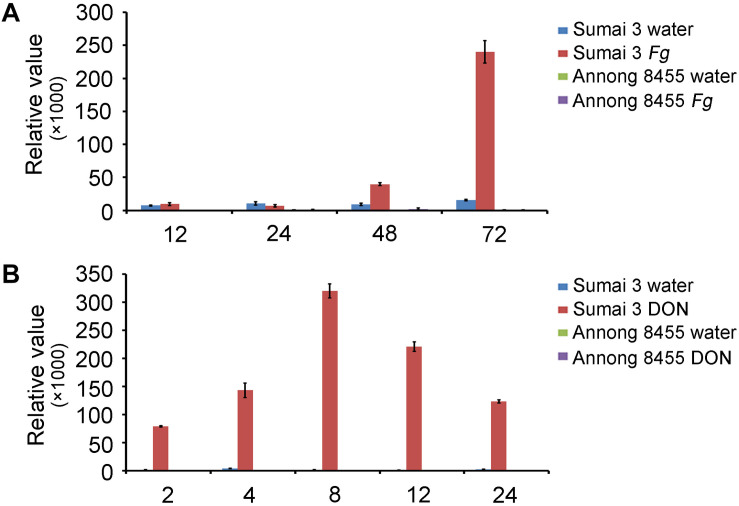
Expression patterns of *TaUGT6* in the glumes of the FHB-resistant wheat cultivar Sumai 3 and the susceptible cultivar Annong 8455 treated with *Fusarium graminearum* **(A)** and deoxynivalenol **(B)**. Samples were collected in a series of hours after inoculation (hai); expression was normalized to tubulin; error bars indicate mean ± standard error of the results of triplicates.

In a previous study, we conducted genome-wide analysis of family-1 UDP-glycosyltransferases in wheat and found many *UGT* genes that were upregulated after *F*. *graminearum* inoculation (He et al., 2018). The novel gene *TRIAE_CS42_5BL_TGACv1_404184_AA1288920* (*TraesCS5B02G436300*), termed *TaUGT6*, was chosen for further characterization in the present study. We firstly performed quantitative reverse transcription polymerase chain reaction (RT-qPCR) to determine the expression level of *TaUGT6* following *F*. *graminearum* inoculation of spikes at different time points in the FHB-resistant cultivar Sumai 3 and susceptible cultivar Annong 8455. *TaUGT6* transcript level was induced at 48 h after *F*. *graminearum* inoculation (hai) and rapidly increased at 72 hai in Sumai 3. This increase was much lower in Annong 8455 (Figure 1A). *TaUGT6* expression level following DON treatment was further analyzed in both Sumai3 and Annong 8455. *TaUGT6* transcript level gradually increased 2 h after DON inoculation (hai) after reaching a peak at 8 hai in Sumai 3, whereas was hardly induced in Annong 8455 treated with DON (Figure 1B).

### Sequence Analysis of *TaUGT6*

**FIGURE 2:**
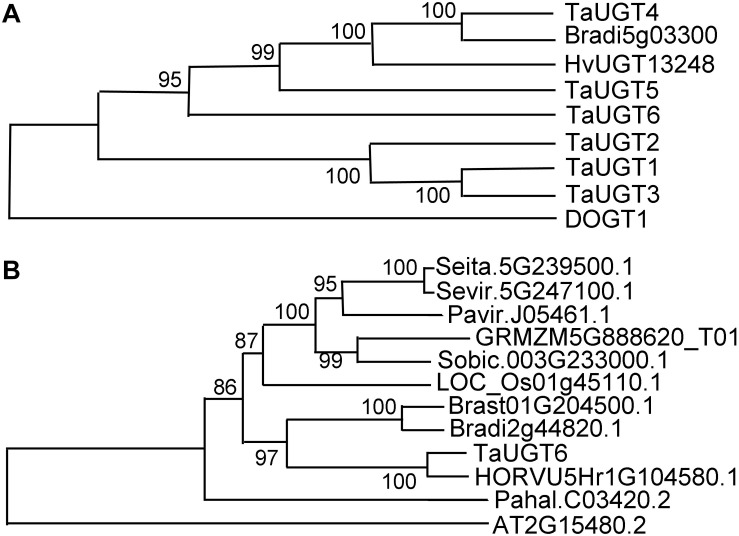
Phylogenetic analyses of TaUGT6 and its close homologs. **(A)** The phylogenetic tree of TaUGT6 and the UGTs previously reported to contribute to FHB resistance. TaUGTs were from *Triticum aestivum*, HvUGT1328 from *Hordeum vulgare*, DOGT1 from *Arabidopsis thaliana*, and Bradi5g03300 from *Brachypodium distachyon*. **(B)** Phylogeny of TaUGT6 and its close homologs. A neighbor-joining tree was constructed by MEGA 7.0 using TaUGT6-related sequences from *Setaria italica* (Seita.5G239500.1), *S*. *viridis* (Sevir.5G247100.1), *Panicum virgatum* (Pavir.J05461.1), *Zea mays* (GRMZM5G888620_T01), *Sorghum bicolor* (Sobic.003G233000.1), *Oryza sativa* (LOC_Os01g45110.1), *B*. *stacei* (Brast01G204500.1), *B*. *distachyon* (Bradi2g44820.1), *H*. *vulgare* (HORVU5Hr1G104580.1), *P*. *hallii* (Pahal.C03420.2), and *A*. *thaliana* (AT2G15480.2).

Based on the public Chinese Spring sequence, full-length *TaUGT6* cDNA encoding a predicted protein of 490 amino acids was amplified. The 1200-bp DNA sequence upstream of *TaUGT6* and the open reading frame were amplified in both Sumai 3 and Annong 8455, and no differences were observed between the two cultivars. The protein sequences of several UGT proteins that had been previously reported to contribute to FHB resistance were aligned by DNAMAN with the pairwise method. The results revealed that TaUGT6 shared a low sequence similarity with TaUGT1/3 (32.94%), TaUGT2 (35.08%), TaUGT4 (22.36%), and TaUGT5 (25.54%) at the amino acid level, respectively. However, all comprised a 44-amino acid UGT consensus sequence, also referred to as PSPG signature box at the C-terminus (Supplementary Figure 1) (Vogt and Jones, 2000). A neighbor-joining phylogenetic tree was constructed using MEGA7.0, and the results showed that apart from *Arabidopsis* DOGT1, TaUGT6 showed high relation to that of known glycosyltransferases, which indicated that they might have a conserved function (Figure 2A).

To further analyze the phylogenetic relationship between TaUGT6 and its close homologs, basic local alignment search tool (BLAST) searches were conducted using the full-length amino acid sequence of TaUGT6 in the Phytozome database, and an unrooted tree was constructed using the neighbor-joining method (Figure 2B). Close homologs of TaUGT6 were found in some grass species, with the closest homolog being barley HORVU5Hr1G104580.1 (Figure 2B).

### Subcellular Localization of TaUGT6

**FIGURE 3:**
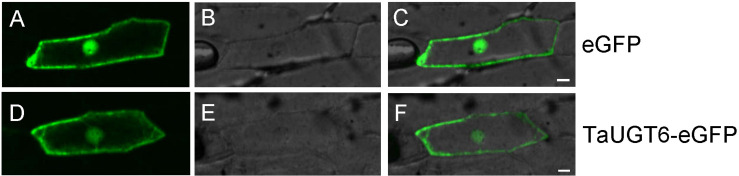
Subcellular localization of TaUGT6 in onion epidermal cells. GFP fluorescence from cells expressing pA7-eGFP vector was distributed throughout cells **(A–C)** and the same in cells expressing TaUGT6–eGFP fusion protein **(D–F)**.

The plasmid pA7-TaUGT6-eGFP was delivered into onion epidermal cells via gene gun to determine the subcellular localization of TaUGT6; pA7-eGFP was used as a control. Fluorescence observation revealed that *TaUGT6* was distributed throughout cells including in cell membrane and nuclei, similar to that in pA7-eGFP (Figure 3). Its subcellular localization was similar to that reported for *TaUGT3* but different from those reported for *TaUGT4* and *TaUGT5* (Ma et al., 2010, 2015; Zhao et al., 2018).

### TaUGT6 Catalyzes DON Glucosylation *in vitro*

**FIGURE 4:**
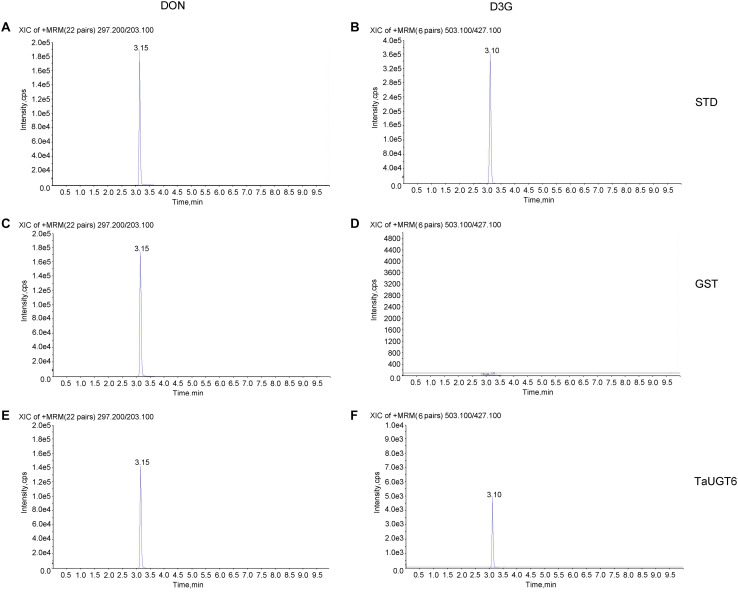
Enzyme activity analysis of TaUGT6 in DON glucosylation. GST or GST-tagged TaUGT6 proteins were added to the reaction mixture with UDP-glucose as the sugar donor and DON as the substrate and analyzed using HPLC–MS/MS. HPLC–MS/MS analysis of the DON standard substance **(A)** and DON-3-O-glucoside standard substance **(B)**. HPLC–MS/MS analysis of DON **(C)** and D3G **(D)** in the reaction mixture with GST after 3 hours reaction. The reaction mixture with GST-tagged TaUGT6 proteins after 3 hours reaction was analyzed, and DON **(E)** and D3G **(F)** were detected.

*TaUGT6* encodes a putative UGT, and its activity has not been previously demonstrated. We then tested its glucosyltransferase activity *in vitro*. Enzyme activity analysis was conducted using UDP-glucose as the sugar donor and DON as the substrate, together with purified GST or GST-tagged TaUGT6 proteins, followed by HPLC–MS/MS. The standards DON and D3G were clearly found at 3.15 min and 3.10 min with m/z 297.200/203.100 for DON (Figure 4A), and m/z 503.100/427.100 for D3G (Figure 4B). D3G was not detected in the reaction mixture containing GST (Figure 4D), but could be observed in the reaction mixture containing GST-tagged TaUGT6 after 3 hours reaction (Figure 4F), which indicated that TaUGT6 was able to transform DON into the less toxic form D3G to some extent (Figure 4E). D3G exhibited a dramatically reduced ability to inhibit protein synthesis and other damage during Fusarium infection, which suggested a potential role of TaUGT6 in FHB resistance.

### *TaUGT6* Overexpression Increases Root Tolerance to DON

**FIGURE 5:**
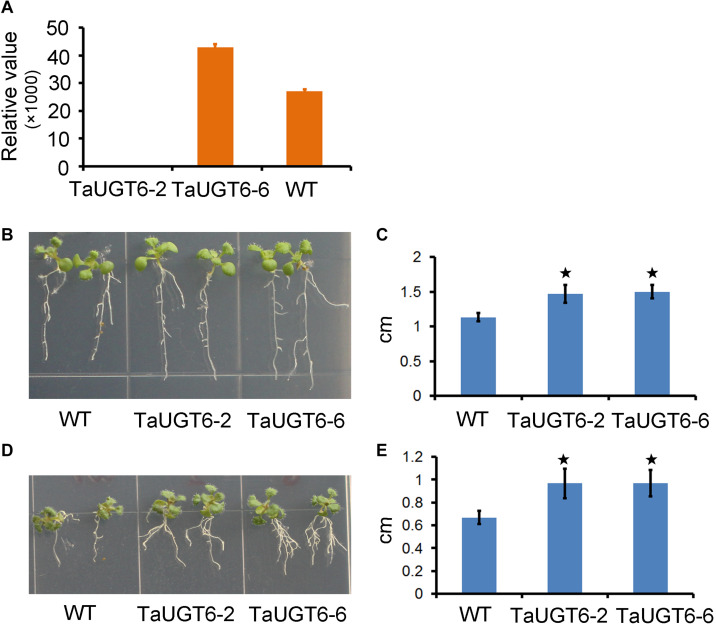
*TaUGT6* overexpression in transgenic *Arabidopsis thaliana* increases root tolerance to DON. **(A)** Relative expression of *TaUGT6* in two overexpressing transgenic lines, i.e., TaUGT-2 and TaUGT-6, and wild-type (WT) Col. **(B)** Seedlings of Col and the transgenic lines after 7 days of growth on MS medium containing 5 ppm DON. **(C)** The longest root lengths measured in **B** (*n* = 10; error bars indicate standard error and stars indicate significant difference; Student’s *t*-test, *P* < 0.001). **(D)** Seedlings after 21 days of growth on MS medium containing 30 ppm DON. **(E)** The longest root lengths measured in D (*n* = 10; error bars indicate standard error and stars indicate significant difference; Student’s *t*-test, *P* < 0.001).

DON reportedly affected root growth on an agar medium (Ma et al., 2010; Pasquet et al., 2016). To determine the involvement of *TaUGT6* and the effects of DON on root growth, we firstly made a construct that allowed the constitutive expression of *TaUGT6* under the control of the tandem 35S promoter, which was transformed into *A*. *thaliana* by the floral dip method. T3 homozygous seeds that had a high expression level of *TaUGT6* from two independent overexpressing transgenic lines (Figure 5A), i.e., TaUGT6-2 and TaUGT6-6, and wild-type (WT) Col were germinated on toxin free medium, and 3-days-old seedlings with the same size and growth vigor were transferred to agar containing 5 or 30 ppm DON. Following 7 days of growth, the phenotype of *Arabidopsis* treated with 5 ppm DON was analyzed. The main toxic effects observed were root growth inhibition (Figure 5B). The transgenic lines showed low inhibitory levels, with the average values for the longest root length in TaUGT6-2 and TaUGT6-6 being 1.47 and 1.5 cm, respectively, compared with 1.13 cm in Col (Figures 5B,C). Root growth was severely inhibited after 21 days of 30 ppm DON exposure, and the longest root lengths of TaUGT6-2 and TaUGT6-6 were significantly greater than that of Col (Figures 5D,E), which indicated that *TaUGT6* overexpression could increase root tolerance to DON accumulation.

### *TaUGT6* Overexpression Enhances Resistance to *Fusarium* Spread

**FIGURE 6:**
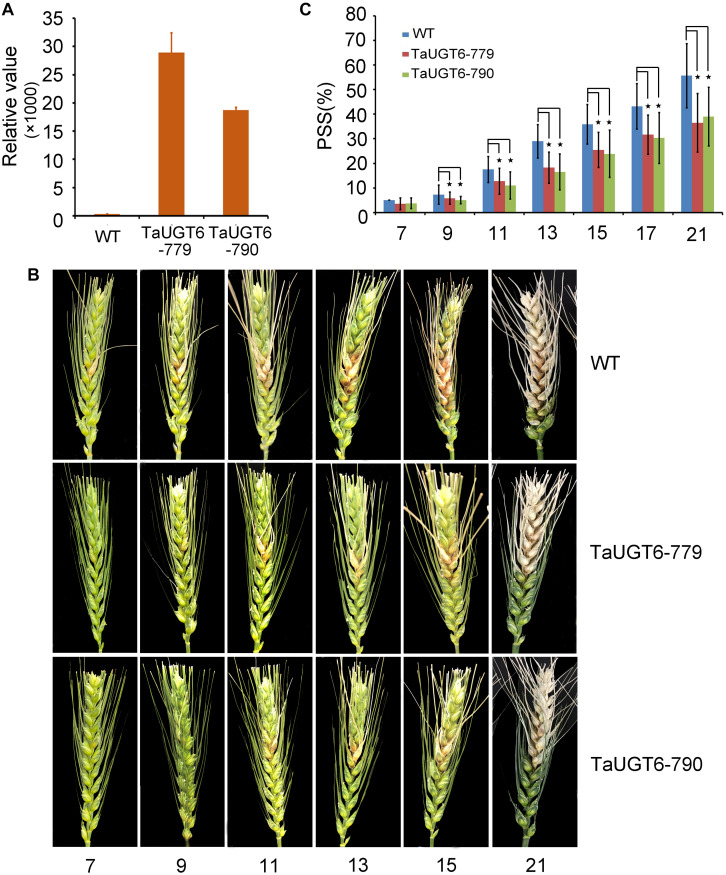
*TaUGT6* overexpression in wheat enhances resistance to *Fusarium* spread. WT and transgenic lines were grown in single-row-plots in greenhouse. **(A)** Relative expression of *TaUGT6* in two overexpressing transgenic lines, i.e., TaUGT-779 and TaUGT-790, and wild-type Fielder (WT). **(B)** Photograph showing typical *Fusarium* spread in spikes of plants from Fielder and the transgenic lines in a series of days after *F*. *graminearum* inoculation. **(C)** Statistical analysis of the proportion of symptomatic spikelet between Fielder and the transgenic lines (*n* = 20, error bars indicate standard error and stars indicate significant difference; Student’s *t*-test, *P* < 0.05).

**FIGURE 7:**
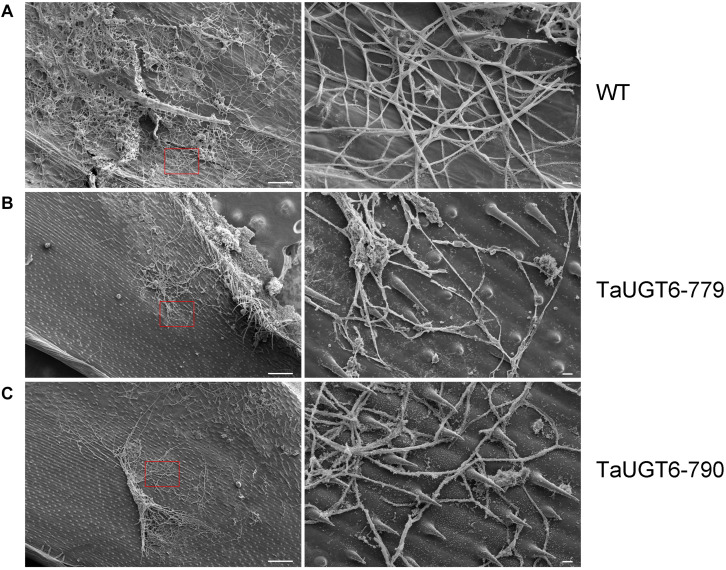
Scanning electron micrographs of wheat spike colonization by *F. graminearum*. **(A)** Hyphal network on the inner surface of the lemma in wild-type Fielder (WT) 3 days after inoculation (dai), with a partial enlargement of the right side (red box). **(B)** Hyphal network in the TaUGT-779 at 3 dai. **(C)** Hyphal network in the TaUGT-790 at 3dai. Bars = 100 μm in the left, and 10 μm in the right.

Investigation of the impact of *TaUGT6* on *F*. *graminearum* infection involved the following. We first transformed *TaUGT6* with a maize ubiquitin promoter into susceptible cultivar Fielder via the *Agrobacterium*-mediated method. Two independent overexpressing transgenic lines TaUGT6-779 and TaUGT6-790 with higher basal transcript level (untreated) than the WT line were chosen for further analysis (Figure 6A). The resistance level of the T3 lines TaUGT6-779 and TaUGT6-790 and the WT Fielder was tested by a single floret inoculation and recorded 7 days after inoculation (dai). No significant difference was found in terms of the proportion of symptomatic spikelet (PSS) between Fielder and the transgenic lines at 7 dai, but TaUGT6 overexpression lines clearly displayed the phenotype less (Figure 6B and Supplementary Figure 2). Furthermore, the overexpressing transgenic lines grown in single-row-plots in the greenhouse showed significantly low PSS from 9 to 21 dai (e.g., the average PSS in TaUGT6-779 and TaUGT6-790 was 36.5% ± 11.9% and 39.1% ± 12%, compared with 55.7 ± 22.3% in Fielder at 21 dai, respectively) (Figures 6B,C). Similar results were found in the overexpression lines grown in plastic pots in the greenhouse (e.g., the average PSS in TaUGT6-779 and TaUGT6-790 was 48.5 ± 14.3% and 46.4 ± 25.4%, compared with 66.1 ± 11.6% in Fielder at 15 dai, respectively) (Supplementary Figure 2). For further exploration of the FHB resistance in the transgenic lines, the infection condition of *F*. *graminearum* in spikes was investigated using scanning electron microscopy. Three days after inoculation, the fungus had developed a dense hyphal network on the inner surfaces of the lemma in the WT Fielder (Figure 7A). However, a less dense hyphal network was clearly seen in both TaUGT6-779 and TaUGT6-790, and the hyphal was not smooth in the WT Fielder (Figures 7B,C). These results showed that *TaUGT6* overexpression could increase FHB resistance in plant tissue.

### *TaUGT6* Overexpression Decreases the Contents of DON

**FIGURE 8:**
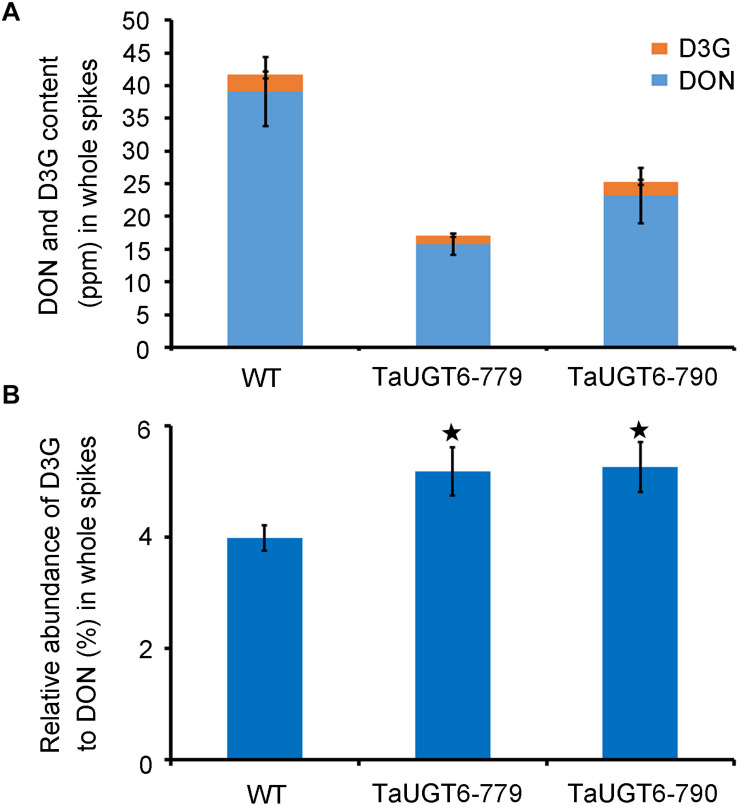
TaUGT6 decreases the contents of DON in planta. WT and transgenic lines were grown in single-row-plots in greenhouse. **(A)** DON (blue bars), and D3G (orange bars) absolute quantification in whole spikes 15 dai. Data represents the mean values of three independent biological replicates; error bars represent the standard deviation. **(B)** D3G molar percentage in “total DON” - D3G/(DON+D3G) in whole spikes 15 dai (error bars indicate standard error, and stars indicate significant difference; Student’s *t*-test, *P* < 0.05).

To test whether the overexpression of *TaUGT6* reduced DON accumulation, DON and D3G contents were measured 15 dai in whole infected spikes in WT and transgenic lines grown in single-row-plots in the greenhouse by HPLC-MS/MS. The DON contents of the lines TaUGT6-779 and TaUGT6-790 were significantly lower than that of WT (P < 0.01) (Figure 8A). The quantity of total DON (DON+D3G) was reduced to 41% and 61% in lines TaUGT6-779 and TaUGT6-790, respectively, in comparison with the control (Figure 8A). Furthermore, the relative abundance of D3G in “total DON” - D3G/(DON+D3G) was 5.18% and 5.26% for TaUGT6-779 and TaUGT6-790, and 3.98% for WT on a molar basis, respectively, indicating a slight but significant increase in the transgenic lines (Figure 8B).

## Discussion

Breeding to improve FHB resistance, particularly DON accumulation resistance, was the most reliable strategy to control FHB and minimize both yield and quality losses, and knowledge regarding the genetic basis underlining wheat–*F*. *graminearum* interaction was incorporated in this study. In the past few years, massive efforts have been made to fight against FHB such as successful cloning of *Fhb1* genes and application of this QTL into practical breeding as well as production of improved cultivars (Buerstmayr et al., 2019). However, the genetic basis of other resistance types, particularly detoxification of DON or enhanced resistance to the toxin, has not yet been elucidated (Gunupuru et al., 2017). Recently, DON glucosylation, catalyzed by UGTs, has drawn the attention of researchers as a potential source of FHB resistance in plants. *Arabidopsis* DOGT1 was the first identified UGT capable of detoxifying DON, and barley HvUGT13248 and its homolog Bradi5g03300 in *B*. *distachyon* were shown to reduce sensitivity to DON and limit FHB spread (Poppenberger et al., 2003; Li et al., 2015; Pasquet et al., 2016). In wheat, few *UGT* genes have been identified and proven to be functional in FHB resistance. *TaUGT1* was repressed, and *TaUGT2* and *TaUGT4* with high similarity to HvUGT13248 were induced after *F*. *graminearum* inoculation, but no further functional analysis was conducted (Lin et al., 2008; Ma et al., 2015). Thus far, only *TaUGT3*, *TaUGT5*, and *Traes_2BS_14CA35D5D* (orthologous to *Bradi5g03300*) in wheat have been transformed into plants to prove their role in FHB resistance and DON tolerance (Gatti et al., 2018; Xing et al., 2018; Zhao et al., 2018). Thus, discovering more *UGT* genes involved in FHB resistance will help elucidate the host resistance mechanisms that confer resistance to DON and enhance DON detoxification mechanisms and will promote the development of more strategies for improving FHB resistance.

Based on our previous phylogenetic analysis, *TaUGT6* was found to be a family 1 *UGT* Group P member, whereas the previously identified members of *TaUGT1*, *TaUGT2*, and *TaUGT3* belonged to Group D and *TaUGT4* and *TaUGT5* belonged to Groups L and E, respectively (He et al., 2018; Zhao et al., 2018). Close homologs of TaUGT6 were only found in grass species, and the closest homolog was barley HORVU5Hr1G104580.1. Moreover, except the conserved PSPG motif at the C-terminus, TaUGT6 exhibited a low sequence similarity with TaUGT1/3 (32.94%), TaUGT2 (35.08%), TaUGT4 (22.36%), and TaUGT5 (25.54%) at the amino acid level, respectively. *TaUGT6* is a novel *UGT* member that might be involved in FHB resistance. *TaUGT6* was highly induced after *F*. *graminearum* or DON treatment in Sumai 3, whereas this was not the case in Annong 8455. Furthermore, no genetic variation was found within the promoter and gene body regions of *TaUGT6* between Sumai 3 and Annong 8455. The different expression pattern after *F*. *graminearum* or DON treatment in these two cultivars might be caused by epigenetic differences such as high DNA methylation in Annong 8455, which is the subject of our ongoing research. Many studies have shown that epigenetic mechanisms have been involved in fine tuning the responses of wheat to environmental stresses (Kong et al., 2020). For example, due to epigenetic events, *TaCYP81D5* conferred salinity tolerance in salinity-tolerant cultivars; however, this was not the case with sensitive cultivars despite their similar sequences (Wang et al., 2019). Plant UGTs have different subcellular locations such as TaUGT3 localized in the plasma membrane and nuclei (Ma et al., 2010), TaUGT4 in the cytoplasm (Ma et al., 2015), and TaUGT5 in the plasma membrane (Zhao et al., 2018). TaUGT6 localized throughout the cells including plasma membrane and nuclei. The existence of TaUGT6 in the cellular membrane is supposed to play a role in prohibition of DON binding to the cellular membrane in the DON resistant wheat varieties or enhancing the tolerance to DON by retarding fungal colonization (Snijders and Krechting, 1992; Ma et al., 2010; Zhao et al., 2018). The function of TaUGT6 localized in the nucleus is still unknown, but further research is needed to clarify whether increased detoxification leads to more residual ribosomal activity allowing better expression of induced defense transcripts into proteins executing FHB resistance or TaUGT6 influence/interact with other proteins in the nuclei to execute the function of FHB resistance and other processes.

The availability of genomic data has been made more convenient to identify new plant UGTs. However, it was difficult to determine which UGTs possessed DON-inactivating capabilities according to phylogenetic-based homology (Osmani et al., 2009). For example, many homologous UGTs of AtUGT73C5 or HvUGT13248 from rice, barley, and *B*. *distachyon* showed no capability of DON detoxification (Schweiger et al., 2013a). Earlier determination of the catalytic activity of UGTs by heterologous expression in *E*. *coli* or yeast and *in vitro* enzyme activity analysis saved time during the identification of new functional UGTs. In this study, we analyzed enzymatic activity with purified GST-tagged TaUGT6 proteins before conducting genetic work. The catalytic activity (0.25 mM DON/2.5 mM UDP-glucose) of TaUGT6 was carried out 3 hours after reaction, and D3G was detected. Determining the optimum reaction conditions may get a better catalytic efficiency. TaUGT6 was able to transform DON into the less toxic form D3G to some extent *in vitro*, which suggested that it might play a potential role in FHB resistance. DON reportedly inhibited root growth when grown on agar medium, and functional DON detoxification of UGTs could reduce this inhibition (Ma et al., 2010; Pasquet et al., 2016). We overexpressed wheat *TaUGT6* in *Arabidopsis* and found that the transgenic lines could increase root tolerance to DON. To date, few *UGT*s have been transformed into wheat. Barley *HvUGT13248* and its homolog *Bradi5g03300* in *B*. *distachyon* were introduced into wheat by different research groups, and all of the data showed that expression of these genes increased FHB resistance and root tolerance to DON in wheat (Li et al., 2015, 2017; Pasquet et al., 2016; Gatti et al., 2019). For wheat *UGT*s, only two of them, i.e., *TaUGT3* and *TaUGT5* were introduced into wheat to functionally confirm their role in FHB resistance (Xing et al., 2018; Zhao et al., 2018). In the present study, for confirmation of the FHB resistance function of *TaUGT6*, it was overexpressed in a wheat cultivar that was highly susceptible to FHB, referred to as Fielder. In the T3 generation, the transgenic lines were grown in plastic pots or in single-row-plots. No significant difference in PSS was found at 7 dai, but weaker phenotype was clearly seen. Lower FHB severity was found from 9 to 15 dai in both conditions. This indicates that *TaUGT6* could increase resistance to *Fusarium* spread. It has been reported that trichothecene toxins underwent biosynthesis as early as 36 h after inoculation, and that rapid detoxification is key to FHB resistance (Kang and Buchenauer, 1999; Li et al., 2017). The transgenic wheat lines constitutively overexpressing *TaUGT6* displayed an unsmooth, less dense hyphal network at 3 dai, and could converted DON to D3G to a certain extent, which led to a slight but significant increase of the relative abundance of D3G to DON at 15 dai. In addition, the total DON accumulation was nearly 50% reduced compared to the control line. This indicates that TaUGT6 is helpful for FHB resistance.

Mycotoxins degradation is a beneficial method to increase FHB resistance, and enzymes that can degrade mycotoxins are urgently required (Ji et al., 2016). *TaUGT6* is a good candidate gene for FHB control. Protein engineering would be a superior method to improve its enzymatic activity. It would also be more beneficial to pyramid it with other resistance QTLs (e.g., *Fhb1*) in a susceptible background in order to achieve a better resistance level in the future breeding.

## Data Availability Statement

The raw data supporting the conclusions of this article will be made available by the authors, without undue reservation.

## Author Contributions

YH and HM conceived the experiments. YH, LW, XL, and LY performed most of the experiments and analyzed the data. PJ and XZ provided analytical tools and assisted in analyzing the data. JQ and GW performed toxin and HPLC-MS/MS analysis. XZ provided project administration. YH, HM, and XZ wrote the article. All authors have read and agreed to the published version of the manuscript.

## Conflict of Interest

The authors declare that the research was conducted in the absence of any commercial or financial relationships that could be construed as a potential conflict of interest.

## Footnotes

1 https://phytozome.jgi.doe.gov/pz/portal.htmaq

2 http://www.ebi.ac.uk/Tools/msa/muscle/

3 http://www.psc.edu/biomed/genedoc/

4 http://www.megasoftware.net/

## Supplementary Material

Supplementary Figure 1 Alignment of the amino acid sequences of TaUGT6 and the UGTs previously reported to contribute to FHB resistance. Red box indicates the PSPG domain. TaUGTs were derived from *Triticum aestivum*, HvUGT1328 from *Hordeum vulgare*, DOGT1 from *Arabidopsis thaliana*, and Bradi5g03300 from *Brachypodium distachyon*.

Supplementary Figure 2 Statistical analysis of the proportion of symptomatic spikelet between WT and the two T3 overexpressing transcript lines in the greenhouse in plastic pots (error bars indicate standard error and stars indicate significant differences; Student’s *t*-test, *P* < 0.05).

Supplementary Table 1 List of primers used in this study.
