# Supplementary figures and images for: TaUGT6, a Novel UDP-Glycosyltransferase Gene Enhances the Resistance to FHB and DON Accumulation in Wheat
Source: Front Plant Sci. 2020 Oct 16;11:574775. doi: 10.3389/fpls.2020.574775 (PMC7596251; doi:10.3389/fpls.2020.574775)

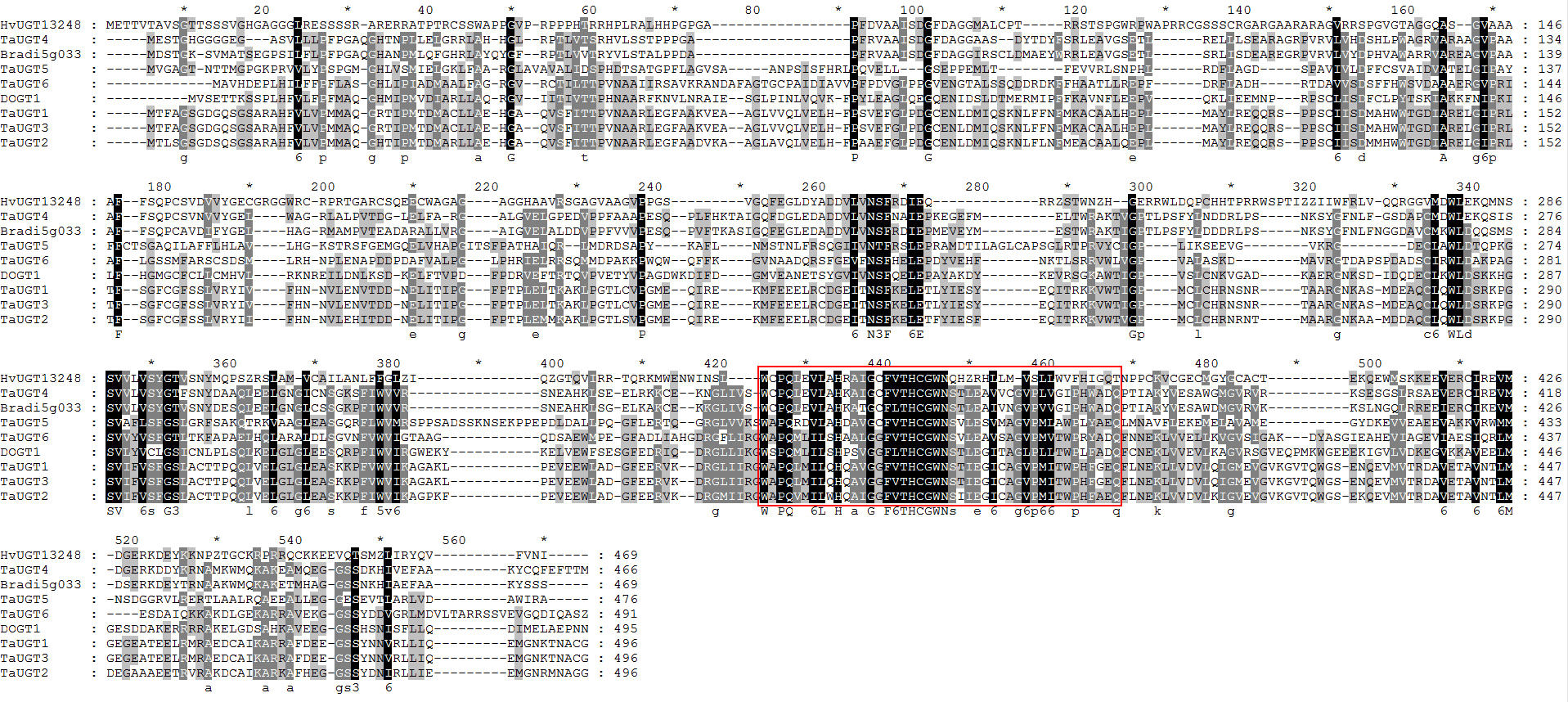

Supplement: Supplementary Figure 1 — Alignment of the amino acid sequences of TaUGT6 and the UGTs previously reported to contribute to FHB resistance. Red box indicates the PSPG domain. TaUGTs were derived from Triticum aestivum, HvUGT1328 from Hordeum vulgare, DOGT1 from Arabidopsis thaliana, and Bradi5g03300 from Brachypodium distachyon. [file Image_1.JPEG]

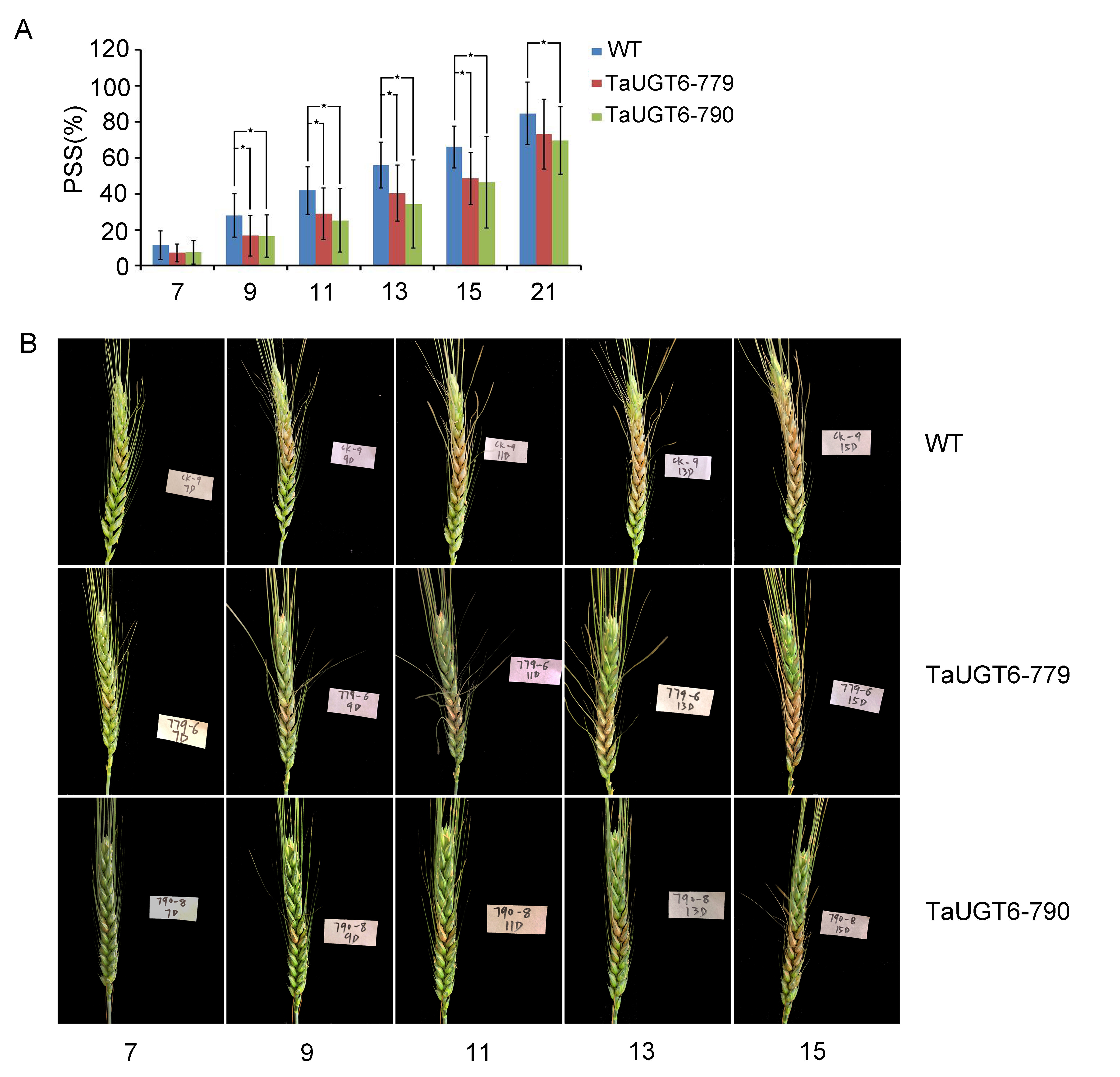

Supplement: Supplementary Figure 2 — Statistical analysis of the proportion of symptomatic spikelet between WT and the two T3 overexpressing transcript lines in the greenhouse in plastic pots (error bars indicate standard error and stars indicate significant differences; Student’s t-test, P < 0.05). [file Image_2.JPEG]
